# A Text-Book of Medical Jurisprudence and Toxicology

**Published:** 1915-06

**Authors:** 


### Reviews of Books.

**A Text-Book of Medical Jurisprudence and Toxicology.** By JOHN GLAISTER, M.D., D.P.H. Camb., F.R.C.S. Pp. xv. 857. Third Edition. Edinburgh: E. & S. Livingstone. Price 15s. net.

A valuable work by an authority on the subject, and, as a book of reference, one which will be of great use to lawyers and medical men. It is comprehensive in its scope, and includes much information with regard to the legal position of the medical practitioner in his relationship with the General Medical Council, the Coroner and the Magistrate.

In discussing the question of conflicting medical evidence the author, referring chiefly to civil actions, very wisely remarks, "While it might be Utopian to expect that medical witnesses for both sides should confer together regarding every kind of cause, there can be little doubt that benefit would result if the witnesses were reasonable men." Such a custom is much to be desired, both in the interests of justice and of the medical profession. Medical evidence and certificates are often regarded by the public as "negotiable" matters, and largely questions of price. The removal of this opprobrium rests entirely with the medical profession; the medical man's position, in a legal case, differs widely from that of a lawyer or advocate; this difference cannot be too much insisted upon.

The illustrative cases, especially those dealing with criminal responsibility, given in the work, are most interesting reading.

From the recital of the Hippocratic oath,—the mention of the Roman law that no pregnant woman was allowed to be interred until the child had been extracted from her womb,—

Abderhalden's Ninhydrin reaction,—down to the record that hot cross buns caused the illness of over a hundred persons in Inverness, nothing appears to have been omitted that may be of interest or value in a work of this kind.

The illustrations are numerous, and are of real assistance to the student.

